# Fabrication Quality Analysis of a Fiber Optic Refractive Index Sensor Created by CO_2_ Laser Machining

**DOI:** 10.3390/s130404067

**Published:** 2013-03-26

**Authors:** Chien-Hsing Chen, Bo-Kuan Yeh, Jaw-Luen Tang, Wei-Te Wu

**Affiliations:** 1 Department of Physics, National Chung Cheng University, Chiayi 621, Taiwan; E-Mails: saesozj@yahoo.com.tw (C.-H.C.); jawluen@gmail.com (J.-L.T.); 2 Department of Biomechatronics Engineering, National Pingtung University of Science and Technology, Pingtung 912, Taiwan; E-Mail: bo.kuang1@gmail.com

**Keywords:** CO_2_ laser machining, optical fiber sensor, refractive index sensing

## Abstract

This study investigates the CO_2_ laser-stripped partial cladding of silica-based optic fibers with a core diameter of 400 μm, which enables them to sense the refractive index of the surrounding environment. However, inappropriate treatments during the machining process can generate a number of defects in the optic fiber sensors. Therefore, the quality of optic fiber sensors fabricated using CO_2_ laser machining must be analyzed. The results show that analysis of the fiber core size after machining can provide preliminary defect detection, and qualitative analysis of the optical transmission defects can be used to identify imperfections that are difficult to observe through size analysis. To more precisely and quantitatively detect fabrication defects, we included a tensile test and numerical aperture measurements in this study. After a series of quality inspections, we proposed improvements to the existing CO_2_ laser machining parameters, namely, a vertical scanning pathway, 4 W of power, and a feed rate of 9.45 cm/s. Using these improved parameters, we created optical fiber sensors with a core diameter of approximately 400 μm, no obvious optical transmission defects, a numerical aperture of 0.52 ± 0.019, a 0.886 Weibull modulus, and a 1.186 Weibull-shaped parameter. Finally, we used the optical fiber sensor fabricated using the improved parameters to measure the refractive indices of various solutions. The results show that a refractive-index resolution of 1.8 × 10^−4^ RIU (linear fitting R^2^ = 0.954) was achieved for sucrose solutions with refractive indices ranging between 1.333 and 1.383. We also adopted the particle plasmon resonance sensing scheme using the fabricated optical fibers. The results provided additional information, specifically, a superior sensor resolution of 5.73 × 10^−5^ RIU, and greater linearity at R^2^ = 0.999.

## 1. Introduction

Various biosensors, such as the electrochemical sensors developed by Clark and Lyons [1], use a galvanometer to measure the glucose concentration of a solution and achieve the measurement objectives. Additionally, semiconductor ion-sensitive biosensors adopt a semiconducting structure that comprises metal-insulating field effect transistors (MISFET) [2]. Another example are the optical fiber sensors that exploit the optical fiber transmission characteristics to achieve sensing objectives, such as evanescent wave and surface plasmon resonance technologies [3].

Of the various biosensor types, optical fiber biosensors offer the unique characteristic of no electromagnetic interference. Small, lightweight, and with the potential for miniaturization, optic fibers can be used not only to transmit light signals, but also as the primary sensing element. Optical fibers are widely employed for engineering and environmental control and in mechanical and biological developments [4].

**Figure 1.**
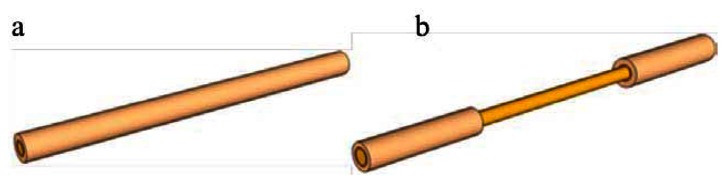
Schematic of the fiber sensor: (**a**) crude fiber; and (**b**) fiber sensors (window type).

Optical fibers have a three-layer structure that comprises a silica-based fiber core, a polymer cladding, and a coating of harder polymer as the outermost layer that protects the fiber. Various methods and structures to provide optical fibers with sensing capabilities have been developed, including fiber Bragg grating [5], fiber-optic interferometers [6], and window-type optical fiber sensors [7]. Among them, window-type optical fiber sensors, as shown in Figure 1, have the simplest structure; only partial removal of the coating material is required to expose the fiber core beneath. Once exposed, the window-type optical fiber structure allows sensors in a test environment to conduct ambient refractive index sensing using the attenuated total reflection (ATR).

The current methods for stripping part of the optical fiber material can be broadly divided into mechanical and chemical methods. The most common of the many mechanical fiber optic stripping methods involve polishing the stripper or fiber [8]. However, the fact that the fiber optic stripper can potentially damage the fiber core presents a significant disadvantage. The fiber polishing method typically requires more expensive equipment, although it does offer high machining accuracy. The chemical method involves the use of various solutions such as sulfuric acid, which was employed by Matthewson [9]. The optical fiber was soaked in sulfuric acid before heating it to between 180 and 200 °C to soften and strip the outer coating material. Nonetheless, etching quality is also difficult to control because a slight error can generate unexpected processing phenomena that affect the sensing quality. Researchers have also employed the flame vaporization technique by exploiting the melting point characteristics of various layers of the optical fiber cable. This technique is used to vaporize the outer cladding material, exposing the glass fiber core. Although easy to process, the processing scope and duration of this method is difficult to control, rendering it unsuitable for extended research [10]. Other research teams have employed lasers and precision lenses on laser processing platforms with a moving mechanism. This method of removal is less time-consuming compared to the other two methods and the parameters are easier to control. Regarding laser processing, the precision lens on the laser processing platform tends to age, which may affect the accuracy of the moving platform and lead to cause experimental errors. This can also lead to inferior processing results, problems such as an inability to correctly remove materials, and/or changes in the material properties and costs of heating the area because of excessive laser energy [11,12]. However, regardless of which method is employed, they all provide the same disadvantage, that is, a lack of comprehensive post-processing quality control procedures.

Based on the above discussion, and to further understand the basic characteristics of window-type optical fiber sensors, we used CO_2_ laser processing as the fiber optic sensor processing method in this study because the laser-processing parameters are convenient to configure and provide a wider range of basic characteristics. Studies of common optical fiber sensors typically investigate only fiber optic sensor fabrication methods or the resolution and sensitivity of back-end sensing applications; the processing quality of the sensing area is rarely examined [13, 14 and 15]. Poor-quality processing, such as over-processing resulting in excessive removal or modification of material, can reduce the sensor resolution and sensitivity, cause light scattering in the sensing area, insufficient sensing power, or functional surface coverage during subsequent surface plasmon resonance (SPR) or particle plasmon resonance (PPR) detection [16,17]. Therefore, the purpose of this study was to eliminate defects or residue from the sensing area of window-type optical fiber sensors. We examined the CO_2_ laser processing results for the sensing area and established a CO_2_ laser processing quality inspection method. Finally, a window-type optical fiber sensor was developed according to the optimal processing conditions identified in this study, and the sensor performance was subsequently verified.

## 2. Experimental Section

**Figure 2.**
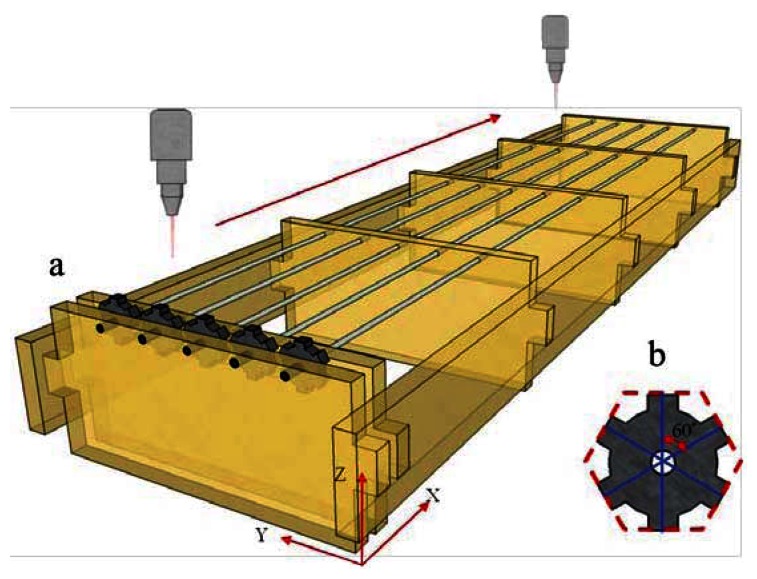
Schematic of the laser machining fixtures: (**a**) the processing fix0ture; and (**b**) the rotating and fixed optical fiber fixture.

The adjustable parameters of the CO_2_ laser machine (Model Mercury-II M-12, LaserPro Inc., New Taipei, Taiwan) employed in this study included the processing power (1 W to 10 W), processing speed (0.63 cm/s to 63 cm/s), laser-sourced cooling nozzle pressure (0.1 MPa at less than 60 psi), and focusing position (adjusted by altering the Z-axis in the machine's three-axis displacement platform). The non-adjustable parameters were the laser pulse width (±0.2 μs) and pulse frequency (5 KHz). This study primarily analyzed the sensing area quality of laser-processed optical fiber sensors. To conduct various quality analyses more accurately, we set the fixed length of the sensing region to 1 cm, the focusing position on the fiber core to 0.1 mm, the laser pulse width to 1 ± 0.2 μs, the laser pulse frequency to 5 KHz, and the air nozzle gauge pressure to 0.3 MPa for air processing. In this study, we considered the laser processing power and speed, in addition to self-developed fixtures, to explore the laser processing path. Figure 2(a) shows the processing fixture used to attach the processed optical fibers; this fixture is capable of attaching five optical fibers simultaneously. Figure 2(b) shows a rotating fixture with a central hole packed tightly with optical fibers ready for processing in lockstep rotation. Using the preset structure highlighted at every 60°, the operator can process optical fibers every 60° a total of six times.

**Figure 3.**
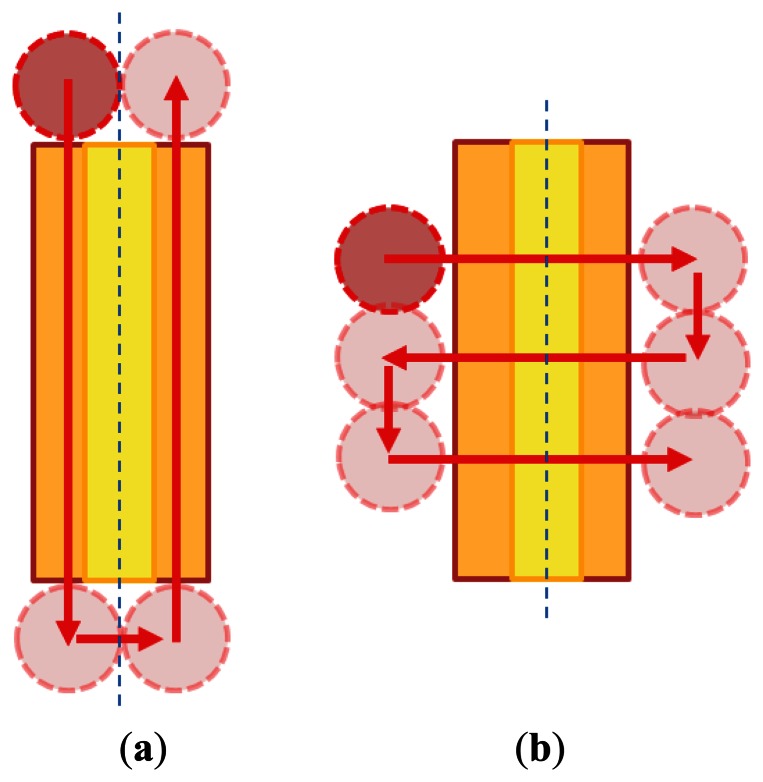
Schematic of the laser processing path: (**a**) parallel machining; and (**b**) vertical machining.

The yellow portion of the structure shown in Figure 3 is the optical fiber to be processed, the blue dotted line denotes the established processing direction of the optical fiber, and the red arrows and circular patterns represent the moving path and processing area of the laser source. When the laser source and the blue dotted reference line move horizontally during processing, the structure adopts the parallel machining condition; otherwise, the vertical machining condition is employed.

**Figure 4.**
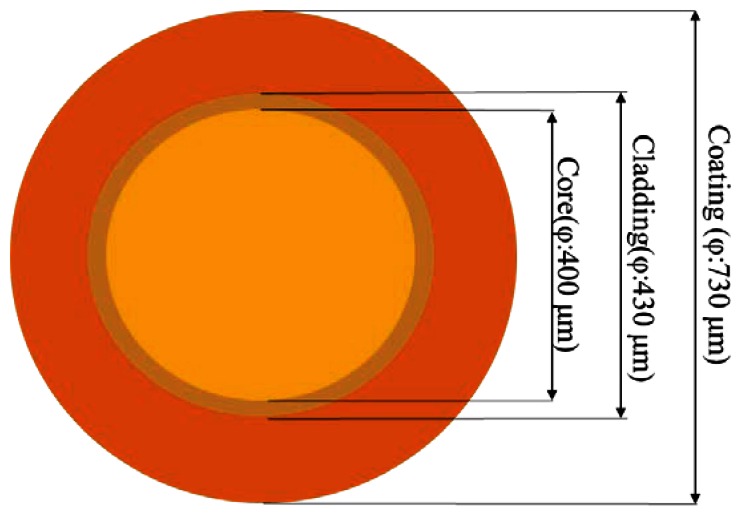
Schematic of the optical fiber.

The research goals of this study were to propose a comprehensive method for assessing the quality of window-type optical fibers processed by lasers, the convenience of light coupling in subsequent sensing measurements, and the mechanical strength of manufactured window optical fibers. Therefore, we employed a multi-mode glass optical fiber with a 400-μm fiber core, manufactured by Newport^®^ under the model number F-MBC, as the optical fiber. Regarding the size and structure, the optical fiber comprised a 400-μm fiber core, 430-μm cladding, and 730-μm coating, as shown in Figure 4. The fiber core was made of silica material, the cladding was made of hard and brittle polymer, and the coating was made of Tefzel material. These materials were used to provide the optical fiber with superior mechanical protection.

**Figure 5.**
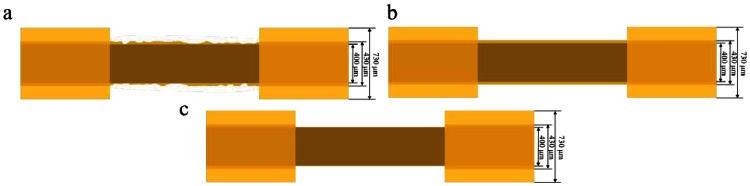
Schematic of an optical fiber cable: (**a**) partially removed; (**b**) insufficiently removed; and (**c**) completely removed.

**Figure 6.**
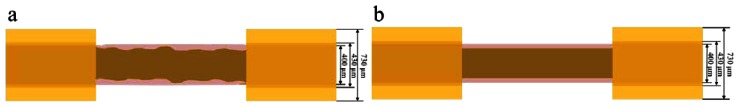
Schematic of optical fiber defects: (**a**) non-uniform changes in property; and (**b**) uniform changes in property.

**Figure 7.**
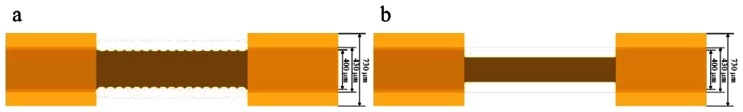
Schematic of an optical fiber: (**a**) wavy pattern; and (**b**) excessive removal.

By adjusting various methods for deploying the laser power, processing speed, and processing path, we defined the following processing defects and circumstances:

1. The fiber core and coating material is either completely or partially removed, as shown in Figure 5.
  (a) The fiber core and coating material are partially removed.
  (b) The fiber core and coating material are insufficiently removed.
  (c) The fiber core and coating material are completely (maybe even excessively) removed.
2. The surface of the fiber core is altered, as shown in Figure 6.
  (a) The surface changes are not uniform.
  (b) The surface changes are uniform.
3. The fiber core shape changes, as shown in Figure 7.
  (a) Wavy pattern
  (b) Excessive removal

**Figure 8.**
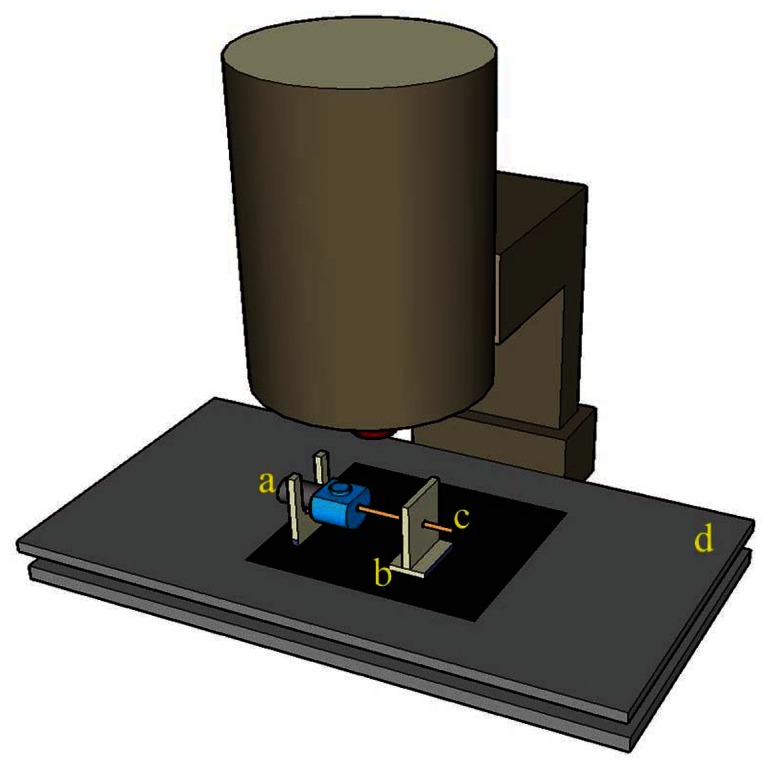
Schematic of the measuring instrument: (**a**) fiber connecter; (**b**) measuring fix0ture; (**c**) processed fiber; and (**d**) micro-stage.

**Figure 9.**
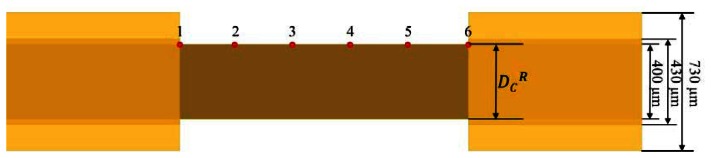
Schematic of the measurement position.

**Figure 10.**
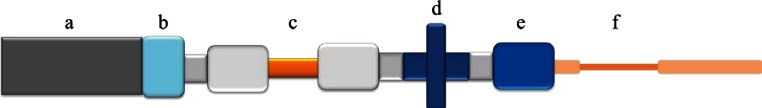
Diagram of the components of an optical light transmission defect: (**a**) laser source; (**b**) collimator; (**c**) fiber cable (NA = 0.27); (**d**) fiber adapter; (**e**) fiber connecter; and (**f**) fiber sensor (NA = 0.37).

**Figure 11.**
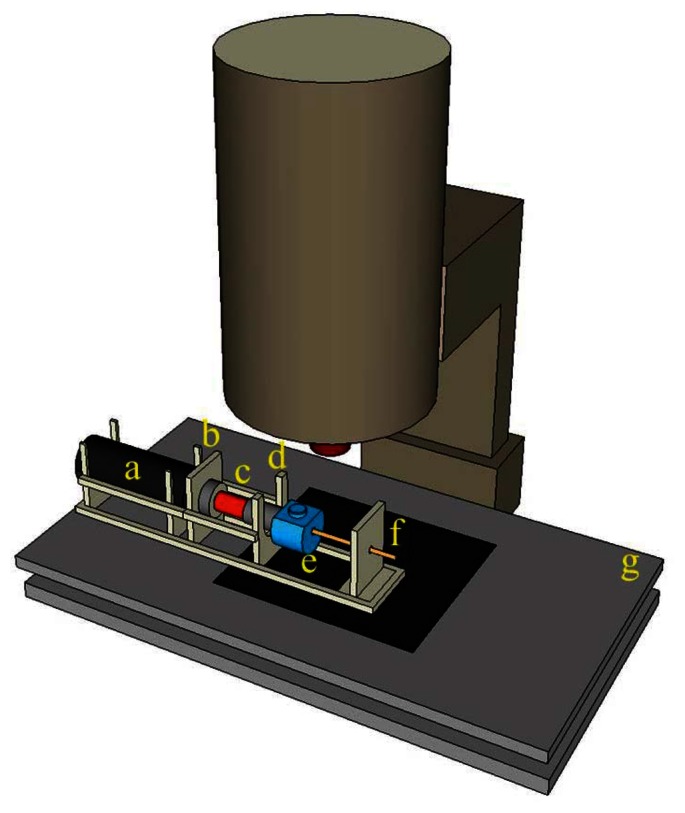
Schematic of the tool used for qualitative analysis of the optical transmission defects: (**a**) laser source; (**b**) collimator; (**c**) fiber cable; (**d**) fiber adapter; (**e**) fiber connecter; (**f**) processed fiber; and (**g**) micro-stage.

**Figure 12.**
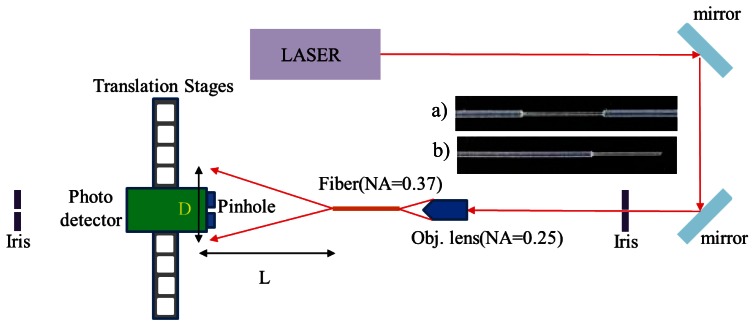
Schematic of the numerical aperture measurement instrument.

**Figure 13.**
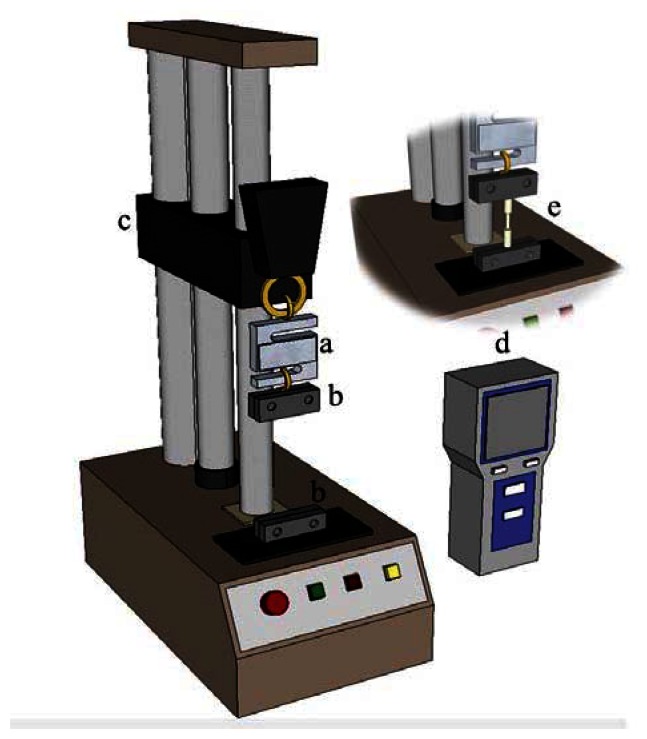
Schematic of the tensile test (Model FS-1002, Lutron Inc., New Taipei, Taiwan): (**a**) force gauge; (**b**) measuring fix0ture; (**c**) translation stage; (**d**) gauge value display; and (**e**) processed fiber.

**Figure 14.**
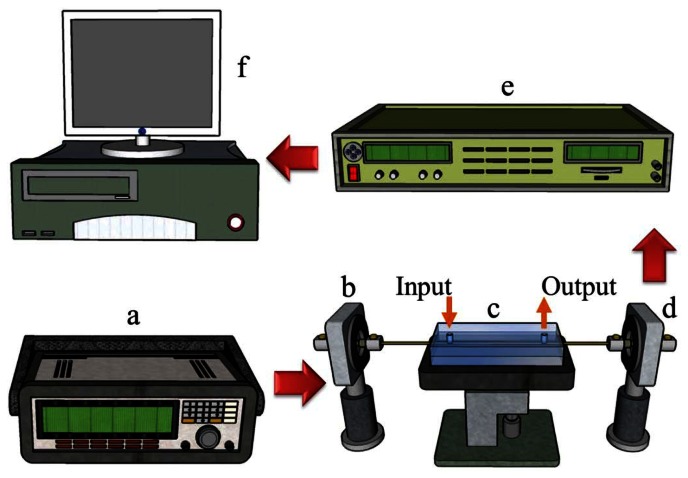
Schematic of the experimental setup for creating sensing measurements: (**a**) function generator (Agilent Inc. Model: 33220A); (**b**) LED light source (Model: EHP-AX08LS-HA/SUG01-P01, Everlight Inc.); (**c**) sensing chip; (**d**) photo diode (Model PD-ET2040, EOT Inc., Traverse, MI, USA); (**e**) lock-in amplifier (Model 7225, Signal Recovery Inc., Oak Ridge, TN, USA); and (**f**) computer.

Therefore, to avoid the defects caused by laser processing, which can affect the sensing capability of optical fiber sensors, negatively influence the sensor performance, or modify other functionalized surfaces, we established a quality analysis method for identifying these defects. This quality analysis method includes the following four items:

(1) Size measurement
  This element can identify lacking, incomplete, or excessive removals and distinguish between the two defect types, namely, wavy pattern and excessive removal, during fiber core modifications. The measuring instrument is shown in Figure 8. The optical fiber sensor location point measurements are shown in Figure 9. First, data from Points 1 and 6 were removed because the cumulative thermal effect at these two points was relatively lower than the processing level in other areas. To prevent the values measured at these two points from affecting the values measured at normal processing areas, we use them to verify the total processing length at the sensing area. Then, the additional diameter of the processing core was used as the average value of the four remaining data groups (the remainder of the core, D_c_^R^).
(2) Light transmission defect detection method
  The light transmission defect detection method was primarily based on internal optical fiber core propagation by optically coupling the laser to the process. For this study, we used the structure shown in Figure 10 to guide the laser light inside the optical fiber sensor as it propagates. First, an optical collimator was used to focus the laser source (wavelength: 532 nm; power: 10 mW) inside the optical fiber transmission line (NA = 0.27) during light propagation. At this time, light from the source was transmitted to the end with an NA value of 0.27 and then guided through the optical fiber adapter as it propagated within the optical fiber core (NA = 0.37). The Optical fiber core was then examined to confirm that the confined light source was guided to the core of the optical fiber when propagating. A diagram of the measurement is shown in Figure 11. When the surface is irregular or contains debris from processing, according to the optical transmission principle, irregular surfaces in the path of light transmissions cause the light to diffuse. Observation of the light diffusion phenomenon facilitates the achievement of quality analysis objectives.
(3) Numerical aperture (NA)
  The two quality analysis methods previous mentioned were used to perform preliminary quality analysis of the laser-processed optical fiber sensors. However, to ensure that no defects remain undetected, additional in-depth investigations of the internal sensing area may be necessary. Therefore, we established a numerical aperture measurement platform to measure the post-processing numerical aperture of optical fiber sensors. The architecture in Figure 12 shows that parallel optical modulation was first conducted. Subsequently, the focusing lens and optical fiber coupler were used to focus and couple the beam onto the core inside the processed optical fiber sensor for light propagation. To measure the NA value of the optical fiber sensor area, we partially cut the processed optical fiber sensor, as shown in Figure 12(a) and 12(b), and employed the grinding method to smooth the sectioned surface for further measurement. After the light coupled to the core layer was propagated, it scattered from the back-end of the truncated plane to the rear, forming a circular light spot with a diameter of D. This was then attached to the optical sensor using the moving platform to measure the size of the circular light spot with a diameter of D at various lengths L. According to Equation (1) [18], the L and D values measured can be employed to determine the numerical aperture value. If the sensing area of the processed optical fiber sensor shows homogeneous surface modification, the NA value of the sensing area decreases. This is because the width of the possible transmission light path is reduced by surface modifications. In this study, we used this method as a basis for identifying defects.
  (1) $$NA=\sin\theta =\frac{D}{\sqrt{D^{2}+4L^{2}}}$$
(4) Weibull tensile test [19,20]
  The Weibull tensile test is suitable for analyzing the tensile properties of hard and brittle materials. The partial exposure of the optical fiber's core silica material during the laser-stripping procedure either alters the material properties or results in internally generated processing defects. If window-type optical fiber sensors contain defects not detected by the previously described quality analysis method, a Weibull tensile test can be employed to exploit the changing trends in tensile failure characteristics. Figure 13 shows a tensile test diagram. Data obtained from the tensile test are analyzed using the Weibull distribution. The mathematical formula for the Weibull distribution is shown as Equation (2), where P is the cumulative probability of failure, σ is the applied tension force (kgf), η is the scale parameter, σ_0_ is the minimum breaking tension force (kgf), and ω is the Weibull modulus:
  (2) $$P=1-\exp[-{\left(\frac{\sigma -\sigma_{0}}{\eta }\right)}^{\omega }]$$
(5) Sensing experiment
  This study investigated whether the processing parameters selected based on the quality analysis results offer sensing capabilities. The setup of the sensing experiment employed for this study is shown in Figure 14. First, a function generator (Model 33220A, Agilent Inc., Santa Clara, CA, USA) was used to generate a direct current pulse with square waves of a 1-KHz frequency and 1-V voltage to drive the green light-emitting diode (LED) light source (Model EHP-AX08LS-HA/SUG01-P01, Everlight Inc., New Taipei, Taiwan). Next, the total reflection characteristic of optical fiber was used to direct the light into the sensing area for ATR sensing. Subsequently, the optical signal was further directed through the optical fiber to the back-end signal capture device for photoelectric signal conversion and processing. The microfluidic chip was made of poly(methyl methacrylate): PMMA (chip size: 50 × 20 × 8 mm; micro-channel size: 50 × 1 × 0.9 mm) and fabricated using a computer numerical control engraving machine by our group [21]. The sensing fiber was packaged inside the microfluidic chip. The input and output ports of the microfluidic chip were used to infuse the solution flowing through the sensing fiber. The sensing environment involved the injection of various sucrose solution concentrations into a sensing microfluidic chip using a syringe for further sensing testing. For the experiment, we first prepared deionized (DI) water. The relationship between the sucrose concentration and refractive index of 1.333 to 1.383 RIU is shown in Table 1 [7]. After the sensing experiment, to compare our results with those reported in previous literature [13], we calculated the resolution of the sensor. Then, we modified the gold nanoparticles in the sensing area to conduct PPR-sensed environmental refractive index measurements [13]. The gold nanoparticles modified in the optical fiber sensing area were prepared by the study researchers [7].

## 3. Results and Discussion

### 3.1. Size Analysis

**Figure 15.**
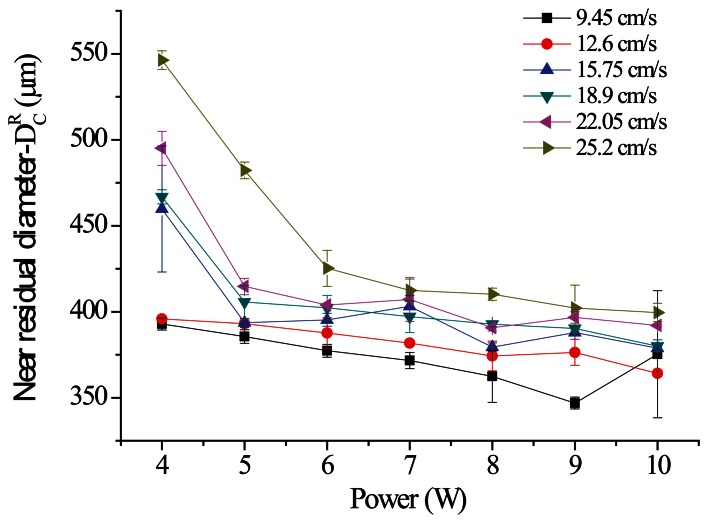
Schematic of the size analysis results (Scanning path: parallel).

**Figure 16.**
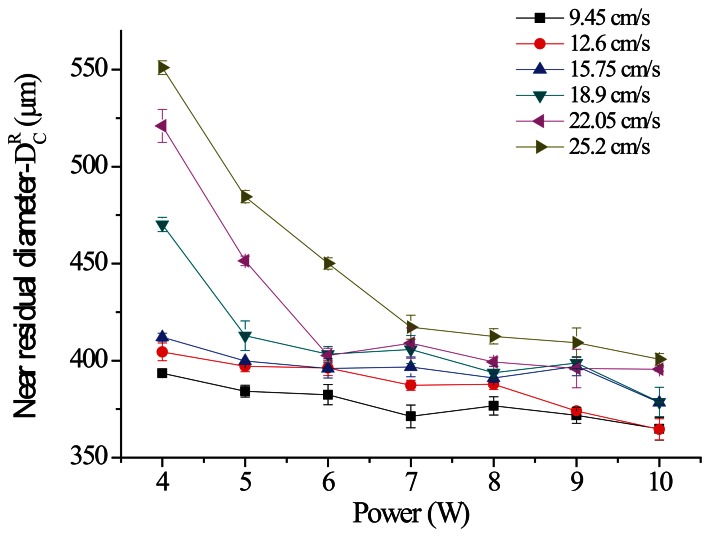
Schematic of the size analysis results (Scanning path: vertical).

This study used the parameter scanning method to identify superior parameters. First, the result of a large-range parameter measurement was employed to obtain a near residual diameter (D_c_^R^) of 400 μm at a processing speed of 9.45 to 25.2 cm/s and to arrange a parameter scanning experiment in this processing speed range. Figure 15 is a diagram showing the relationship between the power of various parallel spindle paths and D_c_^R^; the results indicate that as the processing power increases, the residual diameter gradually decreases under various processing speeds. From the same perspective of processing energy, as the processing speed increases, the total energy loss per unit area per unit of time causes D_c_^R^ to gradually increase. Figure 16 is a diagram of the vertical spindle processing relationship, which exhibits the same conditions. The line graph results in Figures 15 and 16 show that the sloping trend of parallel spindle processing exceeds that of vertical spindle processing. This suggests that the material removal processing change rate under horizontal conditions is comparatively greater. In addition, the standard deviations of the data points from the two processing paths show that parallel spindle path processing is greater than vertical spindle path processing. The cause of this phenomenon may be the greater horizontal processing path displacement, because cumulative structure errors can lead to inconsistent processing quality.

To ensure a residual diameter of nearly 400 μm using laser processing while avoiding the non-processed removal of material from the fiber core layer, we established an acceptable range for the residual diameter:

395μm<(average value+|standard deviation|)<400μm

This range was used to select the following parameters for the second quality analysis process, as shown in Table 2.

### 3.2. Qualitative Analysis of Optical Transmission Defects

**Figure 17.**
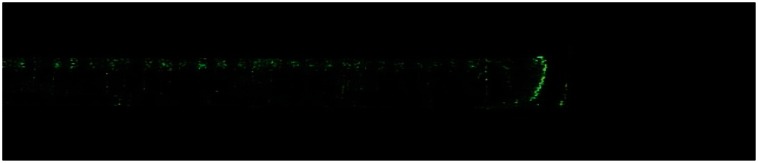
Schematic of the wavy pattern defect.

**Figure 18.**
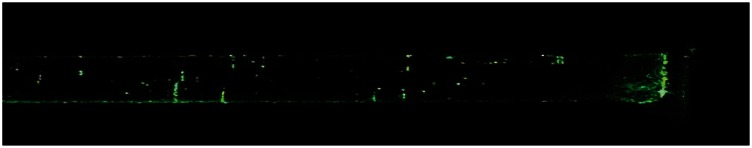
Schematic of the incomplete removal defect.

**Figure 19.**
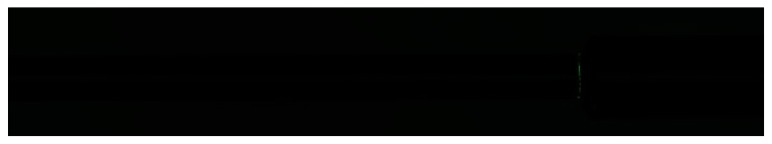
Schematic without any obvious optical transmission defect.

The nine sets of data obtained through size measurements satisfy the criteria for superior parameters (within the expected range). The optical defect transmission method was used to qualitatively analyze the quality of the processing area. The results in Table 3 show that under parallel processing machining conditions, with a laser processing power of 4 W and a processing speed of 9.45 cm/s, excessively low processing speeds result in exorbitant removal of material, generating wavy patterns on the surface of the processing area, as shown in Figure 17. Additionally, regarding the vertical processing machining condition, for optical fiber sensors processed with a power of 5 to 10 W and a speed of 12.6 to 22.05 cm/s, some of the material could not be removed correctly, resulting in the optical leakage phenomenon, as shown in Figure 18. Figure 19 is a diagram of the schematic without defects.

### 3.3. Numerical Aperture Measurement

Preliminary analysis of the quality of laser-processed optical fiber sensors was conducted using two quality analysis methods. We then established an NA measurement platform to measure the NA of the processed optical fiber sensors. During the experiment, we measured the NA of preprocessed optical fibers, obtaining an average value of 0.385 ± 0.01. Compared to the factory specification of 0.37 ± 0.02 with *α* = 0.01, the average value obtained using the two quality analysis methods did not differ significantly. Therefore, the measurement platform developed in this study is feasible.

After conducting the above experiment, we used the quality analysis results to perform separate NA measurement experiments. The results are shown in Table 4 and are relatively small for several NA groups. Because the refractive index between the fiber coating and core layers was modified, according to Equation (3), where n_cladding_ decreases and n_core_ remains the same, the NA value of the material increases [18]. The F-test in an analysis of variance (ANOVA) was used to analyze each group at a significance level of α = 0.05. Using the right-tailed test method with an F significance value as the determination basis, we found that a significant relationship existed between Groups 1, 2, and 3. The value of Group 4 was relatively small, whereas that for Group 5 was large in comparison to the other groups. This result was included in the next quality analysis process to identify defects:

(3) $$NA\equiv \sin\theta_{c}=\sqrt{{n_{\text{core}}}^{2}-{n_{\text{cladding}}}^{2}}$$

### 3.4. Weibull Tensile Test

**Figure 20.**
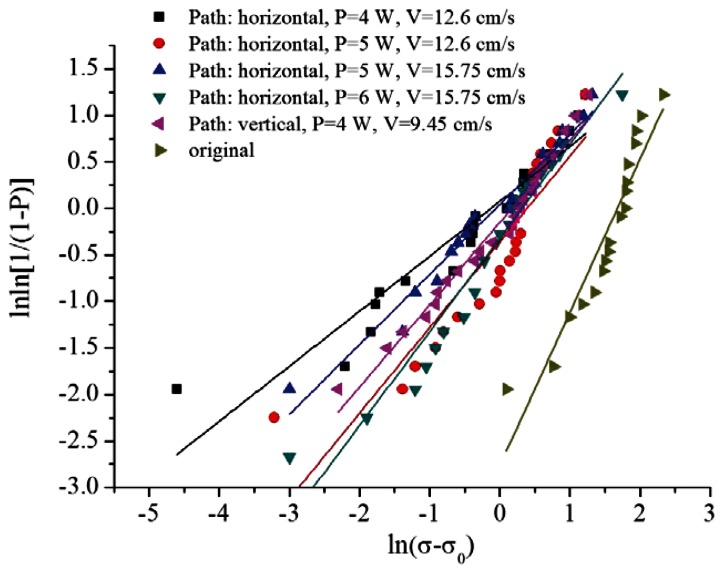
Weibull distribution.

By converting the diagram of stress and cumulative damage probability created using the Weibull tensile test, we can plot a Weibull graph. Figure 20 shows that the information in Table 5 can be obtained after linearly fitting the five parameter groups. The larger the Weibull modulus equation ω in the Weibull distribution, the greater the reliability. Another important parameter is η, which belongs to the scale parameter. The larger this value, the greater the damage to the material. Additionally, the wider the fracture stress distribution, the less homogeneous the material. By contrast, the smaller the value, the more homogeneous the material. Therefore, the resulting table shows that Group 4 has a superior Weibull modulus. However, regarding the results of NA measurements, this group had the smallest value. Thus, to prevent defects, Group 4 was ignored. Statistical analysis of the NA measurement results indicated that Groups 1, 2, and 3 had no significant differences. However, the Weibull tensile analysis results showed that various parameters changed excessively. To avoid the occurrence of non-detected defects, these groups were also eliminated. Therefore, the parameters of Group 5 were selected as the optimum processing parameters for this study.

### 3.5. Refractive Index Sensing Measurements

**Figure 21.**
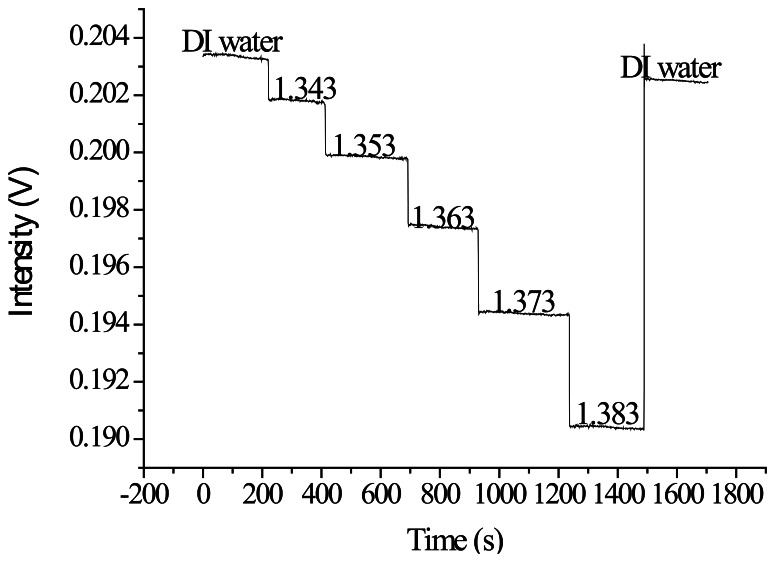
Plot of the ATR fiber sensors' temporal responses to injections of increasing sucrose solution concentrations and refractive indices.

**Figure 22.**
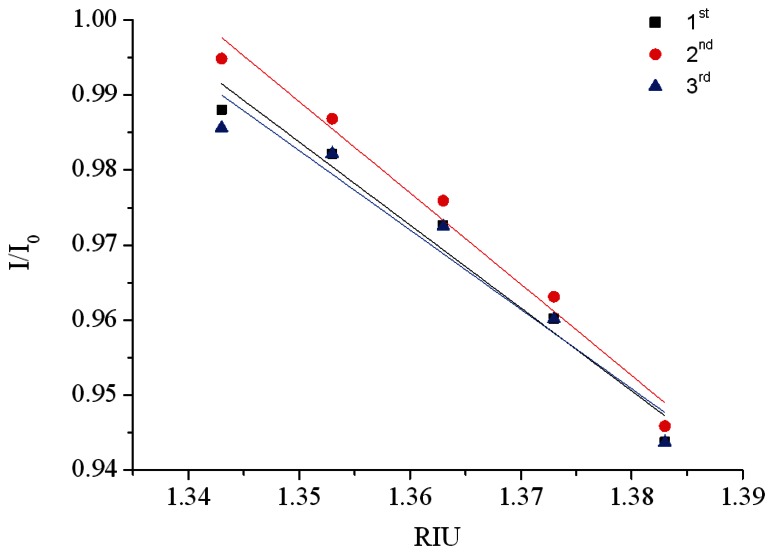
Plot of the ATR fiber sensor response *versus* the refractive index of the sucrose solution.

The quality inspection method previously described was used to select processing parameters for the vertical spindle processing path. A superior set of parameters, including a processing power of 4 W and a processing speed of 9.45 cm/s, was used to create optical fiber sensors for the refractive index sensing experiments. In Figure 21, the X-axis represents the time and the Y-axis represents the signal. Figure 21 shows that the experimental intensity decreased as the injection concentration increased (refractive index increment). When the signal measurement was complete, the sensor resolution was calculated. In Figure 22, the X-axis represents the refractive index value and the Y-axis represents the signal level of each concentration after the average signal was normalized. The relationship diagram in Table 6 shows the sensor slope (m) used to calculate the sensor resolution. After repeating the experiment three times, we obtained an average sensor resolution value of 1.8 × 10^−4^ RIU.

**Figure 23.**
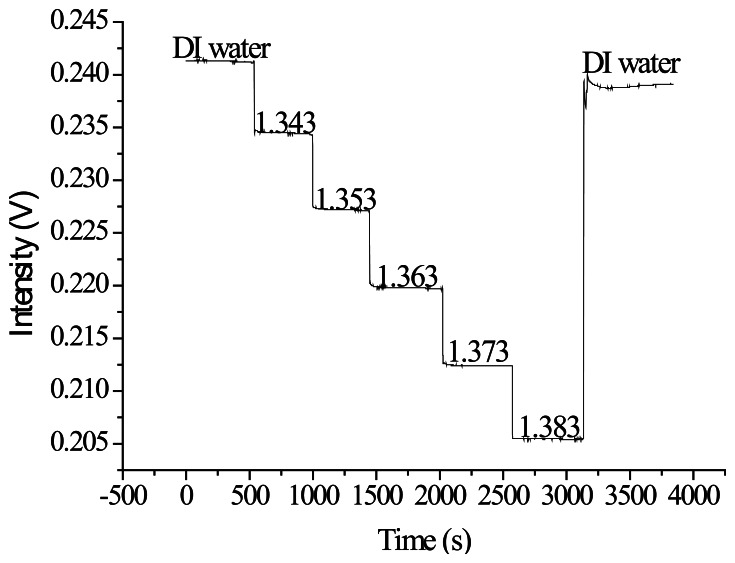
Plot of the PPR fiber sensors' temporal response to injections of increasing sucrose solution concentrations and refractive indices.

**Figure 24.**
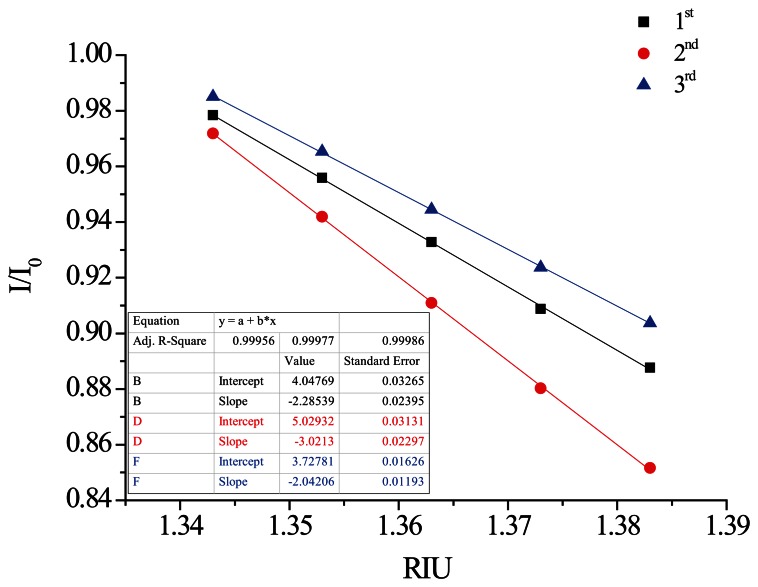
Plot of the PPR fiber sensor response *versus* the refractive index of the sucrose solution.

**Figure 25.**
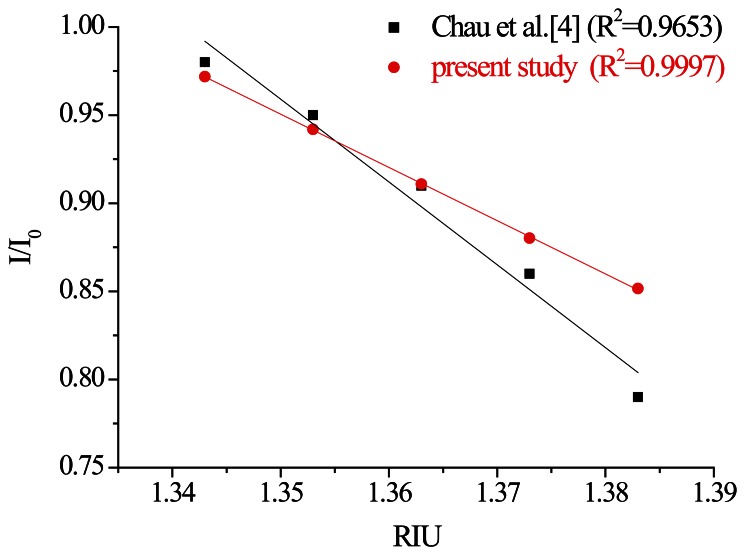
Plot of the sensor response *versus* the refractive index of the sucrose solution.

This study also used the superior processing parameters obtained through quality analysis to create window-type optical fiber sensors by modifying gold nanoparticles [13]. The PPR sensing method was used to conduct three iterations of the refractive index sensing experiment. A diagram of the relationship between the experiment time and signal is shown in Figure 23. The relationship between the average intensity of the refractive index of a measured signal and the refractive index is shown in Figure 24. The results of the sensing resolution after linear regression are shown in Table 7. The average sensor resolution was approximately 5.73 × 10^−5^ RIU. Compared to unmodified ATR sensors, the resolution of the sensor was an order of magnitude smaller as that reported in previous literature, although the sensor length was similar [13]. The results of the linearity comparison modified sensing method are shown in Figure 25. The quality analyzed optical fiber sensors have a superior degree of linearity compared to the non-quality analyzed optical fiber sensors reported in previous studies. This may be because the removal of optical fiber material could not be confirmed in related literature. Thus, quality-analyzed window-type optical fiber sensors possess basic ATR sensing abilities, with the sensor resolution reaching a 10^−4^ RIU level. Once the modified gold nanoparticles sensed using the PPR method were excited, they created a regional plasma resonance response that effectively increased the sensor resolution and linearity.

## 4. Conclusions

The original optical fiber structure of window-type optical fiber sensors has changed according to current sensing needs. The current structure exposes the fiber core to facilitate contact with the sensing environment, and is adopted to measure the environmental refractive index according to the principle of gradually reducing the total reflective sensing. The partial removal method selected for the optical fibers used in this study was the laser thermal removal method. Although adjusting the parameters using this method is extremely easy, because of the thermal effects of removal, if the parameters are not appropriately controlled, defects are likely to result, such as insufficient removal, excessive removal, or property changes. The purpose of removing material is to ensure the core layer of the optical fiber contacts the environment directly. However, if defects exist and the refractive index does not match as expected, the sensitivity of optical fiber sensors may be reduced. The sensing principle employed in this study was the ATR sensing method because the penetration depth of the evanescent wave is extremely limited. In addition, the evanescent wave decays exponentially into the surrounding media because of its distance from the fiber core. However, if other defects exist, the sensing ability is inevitably reduced. In this study, we proposed four methods for analyzing the laser processing quality, that is, size measurements, light transmission defect detection, NA measurement, and a Weibull tensile test. They were employed to verify the quality of the sensing area of optical fiber sensors after laser processing. These methods can also facilitate coating the functional surface, thereby increasing the surface coating rate and sensor sensitivity.

After quality analysis, the remaining processed sensor region measured 400 μm and did not possess light transmission defects. The NA value of the sensing area was 0.52 ± 0.019, with superior processing parameters; that is, a Weibull tensile tested module number ω = 0.886, scale parameter η = 1.186, minimum destructive pulling force of 0.25 kgf, and maximum destructive pulling force of 4.15 kgf. The processing parameters also include a vertical processing path with a processing power of 4 W and a processing speed of 9.45 cm/s. The parameter-processed optical fiber sensors have ATR refractive index sensing capabilities. The average sensor resolution measured was approximately 1.8 × 10^−4^ RIU (R^2^ = 0.954), with the PPR sensing resolution reaching 5.73 × 10^−5^ RIU (R^2^ = 0.999).

The above discussion indicates that in this study, we successfully established a quality analysis method for laser-processed optical fiber sensors. The detection method provides superior quality verification of fiber optic sensors before application by including a quantitative analysis of size, NA, and material properties to eliminate any concerns regarding reduce ATR sensing capability or insufficient functional surface coverage.

## Figures and Tables

**Table 1.** Refractive index of various sucrose solution concentrations [7].

	**RIU**	**Wt %**


**DI water**	1.333	0

**No.1**	1.343	6.8
**No.2**	1.353	13.25
**No.3**	1.363	19.45
**No.4**	1.373	25.4
**No.5**	1.383	31.05

**Table 2.** Size analysis access parameters.

**Scanning Path**	**Power (W)**	**Velocity (cm/s)**	**Avg. D_C_^R^(μm)**	**Std. D_C_^R^(μm)**

Parallel	4	9.45	392.9	3.48
	4	12.6	395.8	0.88
	5	12.6	393.1	2.18
	5	15.75	393.6	2.51
	6	15.75	395.3	3.75

Vertical	4	9.45	393.5	1.77
	5	12.6	397.1	2.77
	8	18.9	393.8	3.36
	10	22.05	395.6	1.12

**Table 3.** Results of the qualitative analysis of optical transmission defects.

**Machining parameter**			**Result**	

**Scanning Path**	**Power (W)**	**Velocity (cm/s)**	**Result**	**Reason**

Parallel	4	9.45	×	Wavy pattern
	4	12.6	O	Without light leakage
	5	12.6	O	Without light leakage
	5	15.75	O	Without light leakage
	6	15.75	O	Without light leakage

Vertical	4	9.45	O	Without light leakage
	5	12.6	×	Removal incomplete
	8	18.9	×	Removal incomplete
	10	22.05	×	Removal incomplete

**Table 4.** Results of numerical aperture measurements.

	**Scanning Path**	**Power (W)**	**Velocity (cm/s)**	**Number of samples**	**NA Avg.**	**NA Std.**
	
	Crude fiber			10	0.385	0.01
		
**No. 1**	Parallel	4	12.6		0.482	0.023
**No. 2**		5	12.6		0.497	0.021
**No. 3**		5	15.75		0.491	0.023
**No. 4**		6	15.75		0.449	0.032
		
**No. 5**	Vertical	4	9.45		0.520	0.019

**Table 5.** Weibull tensile test results.

	**Machining parameter**			**Number of samples**	**ω**	**η**	**σ_min_ (kgf)**	**σ_max_ (kgf)**	**R^2^**

	**Scanning Path**	**Power (W)**	**Velocity (cm/s)**						

**No. 1**	Parallel	4	12.6	30	0.591	0.878	0.14	3.95	0.913
**No. 2**		5	12.6		0.922	1.476	0.25	4.05	0.833
**No. 3**		5	15.75		0.753	0.942	0.1	4.1	0.969
**No. 4**		6	15.75		1.008	1.368	0.1	5.9	0.963
					
**No. 5**	Vertical	4	9.45		0.886	1.186	0.25	4.15	0.974

**Table 6.** The sensing experiment results for ATR fiber sensor.

**No.**	**Sensing slope (m)**	**Coefficient of variation (σ = Standard of deviation/m)**	**RI resolution 3 σ/m (RIU)**

**1**	1.106	7.65 × 10^−5^	2.08 × 10^−4^
**2**	1.217	4.23 × 10^−5^	2.23 × 10^−4^
**3**	1.058	1.07 × 10^−5^	1.09 × 10^−4^

		Average sensor resolution values	**1.8 × 10^−4^**

**Table 7.** Results of the sensing experiment for PPR fiber sensor.

**No.**	**Sensing slope (m)**	**σ value**	**RI resolution 3σ/m (RIU)**

**1**	2.285	1.07 × 10^−5^	1.41 × 10^−5^
**2**	3.021	6.15 × 10^−5^	6.1 × 10^−5^
**3**	2.042	6.6 × 10^−5^	9.69 × 10^−5^

		Average sensor resolution values	**5.73 × 10^−5^**
